# Brief report: Opioid withdrawal in patients with cirrhosis: A retrospective cross‐sectional United States analysis

**DOI:** 10.1111/ajad.70066

**Published:** 2025-07-08

**Authors:** Christopher Behrend, Alexandra D. Ferreira, Spencer R. Goble

**Affiliations:** ^1^ Department of Medicine Hennepin Healthcare Minneapolis Minnesota USA; ^2^ Department of Medicine University of Minnesota Medical School Twin Cities Campus Minneapolis Minnesota USA; ^3^ Department of Medicine, Division of Gastroenterology, Hepatology, and Nutrition University of Minnesota Minneapolis Minnesota USA

## Abstract

**Background and Objectives:**

Patients with cirrhosis face poor outcomes for numerous acute pathologies, but opioid withdrawal outcomes in this population are unknown.

**Methods:**

We conducted a retrospective cross‐sectional study of 306,455 hospitalizations for opioid withdrawal within the National Inpatient Sample from 2016 to 2022.

**Results:**

Patients with cirrhosis (*n* = 2415) had increased rates of mechanical ventilation (aOR = 2.34, *p* = .004), death (aOR = 4.24, *p* = .022) and cost (mean $7845 vs. $4409, *p* < .001).

**Conclusion and Scientific Significance:**

This is the first large, generalizable study of opioid withdrawal in cirrhosis, showing worse outcomes and underscoring the need for careful monitoring in this population.

## INTRODUCTION

With an estimated 5.7 million individuals in the United States affected by opioid use disorder, opioid withdrawal is an important syndrome to monitor and manage.^1^ Symptoms of opioid withdrawal include myalgias, nausea, diarrhea, and autonomic hyperactivity.^2^ Treatment aims to alleviate symptoms using opioid receptor agonists and nonopioid adjunctive medications.^3^ While complications of opioid withdrawal are relatively uncommon, they can occur, particularly in patients with comorbid conditions.^3^

Hepatitis C virus (HCV) and alcohol, two common causes of cirrhosis, are both associated with opioid use disorder and subsequently, patients with cirrhosis have increased rates of opioid use disorder.^4^, ^5^ Additionally, patients with cirrhosis are prescribed opioids more frequently than those with other chronic conditions, such as congestive heart failure and chronic obstructive pulmonary disease.^6^ These high rates of exposure to opioids and opioid use disorder place this population at risk for opioid withdrawal. Unfortunately, cirrhosis is associated with poor outcomes in various acute conditions.^7^ Indeed, acute medical conditions can often precipitate serious complications of cirrhosis such as hepatic encephalopathy and hepatorenal syndrome.^8^, ^9^ Cirrhosis can also significantly alter medication metabolism and places patients at risk for medication adverse effects.^10^ Opioids themselves are a known precipitant for hepatic encephalopathy.^10^ Despite this population's high risk for opioid use disorder and opioid withdrawal along with their tendency for poor outcomes in acute care settings, opioid withdrawal outcomes in this population remain underexplored. This study aims to investigate the outcomes of opioid withdrawal in patients with cirrhosis.

## METHODS

This is a retrospective cross‐sectional analysis utilizing the National Inpatient Sample (NIS) from 2016 to 2022. The NIS is an all‐payer inpatient database that includes approximately 20% of all admissions within the United States. The NIS uses a systematic stratified sampling design to create a data set that, when weighting is applied, creates United States national estimates. Weighting procedures are standardized and provided by the NIS. Weighting was used for this study and results represent national estimates. Included within the NIS is a single primary diagnosis for each admission. This is considered the diagnosis that was chiefly responsible for the admission. Up to 39 secondary diagnoses can be supplied for any given admission and these can represent chronic problems, present on admission diagnoses, or complications of the assessed hospitalization. The NIS is a deidentified, publicly available database and Institutional Board Review approval was waived.

All adult hospitalizations with a primary diagnosis of opioid withdrawal defined as the ICD‐10 codes F11.23, F11.93 were included. Baseline clinical and demographic data were compared between those with and without cirrhosis. The presence of cirrhosis was determined by the presence of a relevant ICD‐10 secondary diagnosis: K71.7, K74.3, K74.4, K74.5, K70.30, K70.31, K74.60, and K74.69. While the NIS does not specify the precise cause of cirrhosis in a patient, secondary diagnoses were used to determine the presence of potential contributing factors such as HCV and alcohol use. Decompensated cirrhosis was also assessed for and was defined as a history of ascites, spontaneous bacterial peritonitis, esophageal varices, hepatorenal syndrome, or hepatic encephalopathy (R18.8, K70.31, K70.11, K71.51, I85.10, I85.00, I85.01, I85.11, K72.91, K72.01, K72.11, K72.90, K76.7, and K65.2). Outcomes were compared between those with and without cirrhosis. The assessed outcomes included: mechanical ventilation, in‐hospital death, mean length of stay (LOS) in days, and mean cost in US dollars. Medians were not assessed due to the weighting that was applied to the data set. Due to suspicion that HCV could confound results, a separate analysis was also performed comparing those with cirrhosis and HCV to those with cirrhosis and no HCV.

Continuous variables were compared using the Student's *t*‐test while categorical variables were compared with chi‐square. Multivariable logistic regression analysis was used to evaluate the associations between cirrhosis and mechanical ventilation and death. Negative binomial regression was used to assess for associations with LOS while a generalized linear model was used to assess for associations with cost. A univariable screen was used to determine the variables included in each multivariable analysis with a *p* < .10 considered the threshold for inclusion. The following were the assessed candidate variables: age, sex, Charlson Comorbidity Index, hospital bed size, and United States hospital region. Due to sample size limitations, only univariable analysis was used in the comparison of HCV‐related cirrhosis to cirrhosis without HCV. All statistical computations were performed using STATA, version 17.0.

## RESULTS

### Participants and descriptive data

A total of 306,455 hospitalization for opioid withdrawal were included, 2415 (0.8%) of which were for patients with cirrhosis. Patients with cirrhosis were older (mean age 53.0 vs. 40.0 years, *p* < .001) and more likely to be male (63.8% vs. 61.6%, *p* < .001) (Table 1). Those with cirrhosis had an increased burden of comorbid disease with a mean Charlson Comorbidity Index of 2.6 compared to 0.5 in patients without cirrhosis (*p* < .001). Patients with cirrhosis also had increased rates of HCV (53.4% vs. 15.5%, *p* < .001), alcohol use (46.8% vs. 17.4%, *p* < .001), and obstructive lung disease (31.7% vs. 15.9%, *p* < .001).

**Table 1:** Characteristics of hospitalizations for opioid withdrawal in patients with and without cirrhosis from 2016 to 2022.

Variable	Cirrhosis	No cirrhosis	*p* Value
Sample size	2415	304,040	
Mean age, years	53.0	40.0	**<.001** **
Female, %	36.2	38.4	**<.001** **
Race, %			
White	67.2	65.8	.530
Black	12.9	19.0	**.002** *
Hispanic	14.6	9.6	**<.001** **
Asian or Pacific Islander	0.6	0.7	.950
Other	4.6	5.0	.712
United States hospital region, %			
Northeast	28.2	32.6	.050
Midwest	20.5	32.0	**<.001** **
South	34.0	23.8	**<.001** **
West	17.4	11.7	**<.001** **
Mean Charlson Comorbidity Index, %	2.6	0.5	**<.001** **
Asthma or COPD, %	31.7	15.9	**<.001** **
Chronic kidney disease, %	7.9	1.9	**<.001** **
Congestive heart failure, %	8.1	1.6	**<.001** **
Obesity, %	8.7	4.1	**<.001** **
Diabetes, %	9.3	3.9	**<.001** **
Hepatitis C virus infection, %	53.4	15.5	**<.001** **
Hepatocellular carcinoma, %	1.5	0.0	**<.001** **
Decompensated cirrhosis, %	20.0	‐	‐
Human immunodeficiency virus, %	4.4	1.5	**<.001** **
Obstructive sleep apnea, %	3.1	2.2	**<.001** **
Mental health secondary diagnosis, %	55.1	53.4	.485
Alcohol use secondary diagnosis, %	46.8	17.4	**<.001** **
Cannabis use, %	11.2	18.2	**<.001** **
Sedative, hypnotic or anxiolytic use, %	14.7	17.9	.070
Stimulant use, %	23.0	33.1	**<.001** **
Homelessness, %	10.4	7.7	**.038** *

*Note*: Bold values indicate statistical significant at *p* < .05.

* *p* < .05

** *p* < .001.

### Clinical outcomes

Mechanical ventilation was required in 1758 (0.6%) hospitalizations. Of those hospitalizations, 70 (4.0%) were for patients with cirrhosis. The following variables on univariable screen were associated with mechanical ventilation with a *p* < .10: age (OR = 1.03, *p* < .001), Charlson Comorbidity Index (OR = 1.30, *p* < .001), larger hospital bed size (OR = 2.28, *p* < .001), and United States hospital region (West OR = 2.99, *p* < .001). Patients with cirrhosis were more likely to receive mechanical ventilation with an absolute risk difference (ARD) of 24.3:1000 admissions (*p* = .004).

A total of 90 deaths were recorded (overall in‐hospital mortality of 0.3:1000 admissions) with 15 (16.7%) of those deaths occurring in patients with cirrhosis. Mortality was higher in those with decompensated cirrhosis compared to compensated cirrhosis (2.1% vs. 0.3%, *p* = .042). The following variables were associated with mortality on univariable screen with *p* < .10: age (OR = 1.08, *p* < .001), Charlson Comorbidity Index (OR = 1.74, *p* < .001), larger hospital bed size (OR = 9.54, *p* = .033), and United States hospital region (South OR = 4.75, *p* = .053). Mortality was significantly increased in patients with cirrhosis (ARD = 6.0:1000 admissions) and the difference remained significant after adjusting for confounding variables (*p* = .022) (Table 2).

**Table 2:** Clinical and healthcare utilization outcomes for hospitalizations for opioid withdrawal in patients with and without cirrhosis.

Outcome	Cirrhosis	No cirrhosis	Adjusted odds ratio (95% CI)	*p* Value
Mechanical ventilation^a^ (per 1000 admissions)	29.9	5.6	2.34 (1.31–4.12)	**.004** *
In‐hospital death^a^ (per 1000 admissions)	6.2	0.2	4.24 (1.26–14.27)	**.020** *

Utilization outcomes^b^	‐	‐	Adjusted^a^ rate ratio (95% CI)	*p* Value
Mean length of stay (days)	5.2	3.8	1.20 (0.96–1.51)	.105
Mean cost (US $)	$7845	$4409	1.20 (1.09–1.33)	**<.001** **

*Note*: Bold values indicate statistical significant at *p* < .05.

Abbreviation: CI, confidence interval.

^a^ Adjusted for age, Charlson Comorbidity Index, hospital bed size, and United States hospital region.

^b^ Adjusted for age, sex, Charlson Comorbidity Index, hospital bed size, and United States hospital region.

* *p* < .05

** *p* < .001.

### Healthcare utilization outcomes

The mean LOS for all admissions was 3.8 days. The following variables were associated with LOS on univariable screen: age (RR = 1.01, *p* < .001), female sex (RR = 1.02, *p* = .044), Charlson Comorbidity Index (RR = 1.06, *p* < .001), larger hospital bed size (RR = 1.12, *p* < .001), and United States hospital region (West RR = 1.13, *p* < .001). LOS was increased in patients with cirrhosis (RR = 1.38, 95% CI: 1.11–1.73, *p* = .004) although the difference did not remain significant after adjusting for confounding variables (*p* = .105).

The mean cost for all admissions was $4435. The following variables were associated with cost on univariable screen: age (RR = 1.01, *p* < .001), female sex (RR = 1.07, *p* < .001), Charlson Comorbidity Index (RR = 1.18, *p* < .001), larger hospital bed size (RR = 1.15, *p* < .001), and United States hospital region (West RR = 1.19, *p* < .001). Cost was increased in patients with cirrhosis (RR = 1.78, 95% CI: 1.59–2.00, *p* < .001) and this did remain significant after adjusting for confounding variables (*p* < .001). Cost per hospitalization day in patients with cirrhosis was $1878 which was significantly higher than in patients without cirrhosis ($1306) (*p* < .001).

### HCV‐related cirrhosis

Of the 2415 hospitalizations in patients with cirrhosis, 1290 (53.4%) were for patients with HCV. Patients with HCV and cirrhosis had similar decompensation rates to those with non‐HCV cirrhosis (21.7% vs. 18.2%, *p* = .346). Those with HCV did not have significantly increased mechanical ventilation (3.1% vs. 2.7%, OR = 1.17, 95% CI: 0.40–3.42, *p* = .777) or mortality (0.8% vs. 0.4%, OR = 1.74, 95% CI: 0.16–19.35, *p* = .651) rates. Similarly, LOS (mean 5.8 vs. 4.6 days, RR = 1.28, 95% CI: 0.87–1.88, *p* = .203) and cost (mean $7900 vs. $7781, RR = 1.02, 95% CI: 0.81–1.27, *p* = .895) were not significantly increased in those with HCV.

## DISCUSSION

Patients with cirrhosis experienced increased rates of mechanical ventilation and death along with increased costs and LOS in this inpatient assessment of opioid withdrawal outcomes. To our knowledge, this is the first study that specifically examines the relationship between cirrhosis and opioid withdrawal inpatient outcomes.

Patients with cirrhosis required mechanical ventilation more than five times as frequently as those without cirrhosis. This is important as intubated patients with cirrhosis have been shown to have poor extubation and survival rates compared to those without cirrhosis.^11^ Additionally, those with cirrhosis also had increased mortality. We suspect that these differences are driven by complications of cirrhosis, such as hepatic encephalopathy which could be triggered by opioid withdrawal itself or by medications used to manage opioid withdrawal.^12^ It is also possible that those with cirrhosis are more susceptible to other adverse reactions, such as respiratory failure, to the medications used to treat opioid withdrawal and these contributed to our findings.

Several potential confounding variables may have also influenced the clinical outcomes and warrant specific mention. Alcohol intoxication and withdrawal can both necessitate respiratory intubation for patients who are unable to protect their airway.^13^, ^14^ As these were all secondary diagnoses, it is unclear how many patients had acute intoxication or withdrawal compared to simply a history of alcohol use, and we therefore opted to not include alcohol use in any multivariable analysis. HCV is also a potential confounder as the increased rate in those with cirrhosis could suggest a higher rate of injection drug use.^15^ However, our comparison of those with HCV and cirrhosis to those with cirrhosis and no HCV showed no difference in outcomes. Also of note, those with cirrhosis had increased rates of obstructive lung pathology which also could have contributed to the increased rates of respiratory failure.

Notably, cost and LOS were also increased in those with cirrhosis, likely reflecting the higher acuity and emphasizing the high healthcare utilization in this population. For many patients with opioid addiction, the option of inpatient versus outpatient detoxification can be considered based on patient preference.^16^ Our findings of worse outcomes for those with cirrhosis emphasize the need for careful assessment to select the appropriate level of care for this population, particularly in those with decompensated cirrhosis.

This study was limited by its reliance on ICD‐10 coding which does not provide important information such as the severity of opioid withdrawal and the etiology of cirrhosis. Several confounders such as alcohol use and obstructive lung pathology may have impacted our results, however, using the Charlson Comorbidity Index in multivariable analysis did allow for the analysis to control for overall comorbid illness burden.^17^ Other outcomes during the hospitalization, such as hepatic decompensation events, may have also confounded the findings. A strength of this study is the large data set from the NIS during 2016–2022 which was analyzed. The large sample size and relatively recent data enhance the reliability and relevance of these findings.

Patients with cirrhosis admitted for opioid withdrawal were found to have increased mortality and morbidity compared to those without cirrhosis in this large, United States nationwide study. Careful monitoring is likely appropriate in patients with cirrhosis going through opioid withdrawal.

## CONFLICT OF INTEREST STATEMENT

The authors declare that there are no conflict of interests.
